# Association between gene expression and neurodegeneration in dementia with Lewy bodies

**DOI:** 10.1002/alz.086838

**Published:** 2025-01-09

**Authors:** Annegret Habich, Janna M. Baumann, Christopher G. Schwarz, Scott A. Przybelski, Matteo Pardini, Francesco Calizzano, Anna Inguanzo, Ketil Oppedal, Frédéric Blanc, Afina Willemina Lemstra, Jakub Hort, Matthias Brendel, Miguel A Ochoa‐Figueroa, Valentina Garibotto, Milica G. Kramberger, andrea pilotto, Brad F. Boeve, Dag Aarsland, Eric Westman, Thomas Dierks, Kejal Kantarci, Silvia Morbelli, Laura E. Jonkman, Daniel Ferreira

**Affiliations:** ^1^ Division of Clinical Geriatrics, Department of Neurobiology, Care Sciences and Society, Karolinska Institute, Stockholm, Sweden, Stockholm Sweden; ^2^ Alzheimer Center Amsterdam, Department of Neurology, Amsterdam Neuroscience, Vrije Universiteit Amsterdam, Amsterdam UMC, Amsterdam Netherlands; ^3^ Mayo Clinic, Radiology, Rochester, MN USA; ^4^ Department of Quantitative Health Sciences, Mayo Clinic, Rochester, MN USA; ^5^ Università degli studi di Genova, Genova, Liguria Italy; ^6^ University of Genoa, Genoa, Liguria Italy; ^7^ Division of Clinical Geriatrics, Centre for Alzheimer Research, Karolinska Institutet, Stockholm Sweden; ^8^ University of Stavanger, Stavanger Norway; ^9^ Day Hospital of Geriatrics, Memory Resource and Research Centre (CM2R) of Strasbourg, Hopitaux Universitaires de Strasbourg, Strasbourg France; ^10^ Alzheimer Center Amsterdam, Neurology, Vrije Universiteit Amsterdam, Amsterdam UMC, Amsterdam Netherlands; ^11^ Memory Clinic, Department of Neurology, Charles University, Second Faculty of Medicine and Motol University Hospital, Prague Czech Republic; ^12^ LMU University Hospital, Munich Germany; ^13^ Linköping University, Linköping Sweden; ^14^ Division of Nuclear Medicine and Molecular Imaging, Geneva University Hospitals, Geneva Switzerland; ^15^ University Medical Centre Ljubljana, Medical Faculty, University of Ljubljana, Ljubljana, Ljubljana Slovenia; ^16^ University of Brescia, Brescia, Italy Italy; ^17^ Department of Neurology, Mayo Clinic, Rochester, MN USA; ^18^ Institute of Psychiatry, Psychology and Neuroscience, King's College London, London UK; ^19^ Division of Clinical Geriatrics, Center for Alzheimer Research, Department of Neurobiology, Care Sciences and Society, Karolinska Institutet, Stockholm Sweden; ^20^ University Hospital of Psychiatry and Psychotherapy Bern, Bern Switzerland; ^21^ Department of Radiology, Mayo Clinic, Rochester, MN USA; ^22^ IRCCS Ospedale Policlinico San Martino, Genova, Liguria Italy; ^23^ Amsterdam UMC, location VUmc, Department of Anatomy and Neurosciences, Section Clinical Neuroanatomy and Biobanking, Amsterdam Netherlands

## Abstract

**Background:**

Patterns of regional atrophy and hypometabolism have been observed in dementia with Lewy bodies (DLB). However, determinants of regional vulnerability to structural and functional neurodegeneration remain largely unexplored. First, we investigated the association between regional gene expression and grey matter volumes in probable DLB patients. Since hypometabolism presumably precedes overt brain atrophy, we additionally investigate the association between regional gene expression and hypometabolism in DLB.

**Method:**

To investigate the association between gene expression and regional volumes, 165 DLB patients along with 165 age‐ and sex‐matched healthy controls from three European centres and the Mayo Clinic (USA) were included. Regional volumes were quantified from MRI using SPM12 in 112 cortical, subcortical, and cerebellar brain regions, and compared between groups, using w‐scores. Regional expression data of seven genes involved in the formation and degradation of pathological protein aggregates (APOE, APP, BIN1, GBA, MAPT, SNCA, TMEM175) was extracted from six healthy donors from the Allen Human Brain Atlas and correlated with regional volumetric w‐scores. To assess the predictive value of regional gene expression on regional volumes we used Gaussian stepwise backwards linear regression including all seven genes as predictors. To investigate the association between gene expression and hypometabolism, we are currently applying the same analysis pipeline to FDG‐PET data of 139 DLB patients from 8 European centres.

**Result:**

Most brain regions showed lower volumes in DLB patients compared to healthy controls, with differences being most pronounced in occipital and parietal lobes. Regional expression of APOE correlated positively with regional volumes in DLB. Conversely, regional expression of MAPT correlated negatively with regional volumes. Both APOE and MAPT gene expression were significant predictors of regional volumes in the regression analysis. None of the other gene expression values was significantly associated with regional volumes in DLB.

**Conclusion:**

Our findings show that regional expression of genes associated with the abnormal accumulation of amyloid and tau, common co‐pathologies in DLB, partially account for the brain atrophy pattern observed in DLB patients. Analyses are ongoing for the hypometabolism pattern. Our finding emphasises the relevance of co‐pathologies in predicting atrophy progression and identifying potential targets for future disease‐modifying treatments.

